# Phenogenon: Gene to phenotype associations for rare genetic diseases

**DOI:** 10.1371/journal.pone.0230587

**Published:** 2020-04-09

**Authors:** Nikolas Pontikos, Cian Murphy, Ismail Moghul, Gavin Arno, Kaoru Fujinami, Yu Fujinami, Dayyanah Sumodhee, Susan Downes, Andrew Webster, Jing Yu, UK Inherited Retinal Dystrophy Consortium, Phenopolis Consortium

**Affiliations:** 1 UCL Genetics Institute, University College London, London, United Kingdom; 2 Institute of Ophthalmology, University College London, London, United Kingdom; 3 Moorfields Eye Hospital, London, United Kingdom; 4 Warwick Medical School, University of Warwick, Coventry, United Kingdom; 5 UCL Cancer Institute, University College London, London, United Kingdom; 6 Laboratory of Visual Physiology, Division of Vision Research, National Institute of Sensory Organs, National Hospital Organization Tokyo Medical Center, Tokyo, Japan; 7 Department of Ophthalmology, Keio University School of Medicine, Tokyo, Japan; 8 Graduate School of Health Management, Keio University, Tokyo, Japan; 9 Division of Public Health, Yokokawa Clinic, Osaka, Japan; 10 Queen Mary University, Mile End Road, Bethnal Green, London, United Kingdom; 11 Oxford Eye Hospital, West Wing, John Radcliffe Hospital, Oxford, United Kingdom; 12 Nuffield Department of Clinical Neurosciences, University of Oxford, John Radcliffe Hospital, Oxford, United Kingdom; German Cancer Research Center (DKFZ), GERMANY

## Abstract

As high-throughput sequencing is increasingly applied to the molecular diagnosis of rare Mendelian disorders, a large number of patients with diverse phenotypes have their genetic and phenotypic data pooled together to uncover new gene-phenotype relations. We introduce Phenogenon, a statistical tool that combines, Human Phenotype Ontology (HPO) annotated patient phenotypes, gnomAD allele population frequency, and Combined Annotation Dependent Depletion (CADD) score for variant pathogenicity, in order to jointly predict the mode of inheritance and gene-phenotype associations. We ran Phenogenon on our cohort of 3,290 patients who had undergone whole exome sequencing. Among the top associations, we recapitulated previously known, such as "*SRD5A3*—Abnormal full-field electroretinogram—recessive" and "*GRHL2* –Nail dystrophy—recessive", and discovered one potentially novel, “*RRAGA*–Abnormality of the skin—dominant”. We also developed an interactive web interface available at https://phenogenon.phenopolis.org to visualise and explore the results.

## Introduction

As DNA sequencing cost decreases, whole exome sequencing (WES) has become prevalent in the molecular testing of individuals with rare Mendelian disorders. This has led to the identification of many variants of unknown pathogenicity and clinical significance, with associated difficulty in variant interpretation. A common practice for variant prioritisation is to search for phenotypically similar disease cases with variants in known genes. Conventionally, this is done by searching databases such as dbSNP [1] and ClinVar [2] for genetic variants, Online Mendelian Inheritance in Man (OMIM) for genes, and targeted disease databases such as RetNet [3] for retinal dystrophy. However, when no candidate genes or variants are found in published cases with a known genetic diagnosis, an alternative solution is to group unsolved cases with similar phenotypes to increase the chances of finding shared genetic variations across genes.

The UK Inherited Retinal Dystrophy Consortium (UKIRDC) successfully applied this approach by whole exome sequencing 365 unsolved pre-screened retinal dystrophy patients from London, Leeds, Oxford and Manchester [4–9]. The WES and phenotype data were deposited as part of our Phenopolis database (www.phenopolis.org) [10], which itself hosts 5122 (as of 2nd February 2019) exomes of patients with a range of disorders such as dementia, Crohn’s disease, seizures and bone-marrow failure (S1 Table).

This unique dataset provided the ideal opportunity to develop a novel statistical analysis tool, Phenogenon, in order to uncover gene-phenotype associations from large and phenotypically diverse cohorts of patients. The complete workflow of Phenogenon is described in (S1 Fig). Phenogenon does not require explicit thresholds for variant filtering, which rely on assumptions of disease prevalence and mode of inheritance, but instead bins genetic variants according to their population frequencies (gnomAD) and predicted pathogenicity (CADD) to produce a two-dimensional heatmap for each gene-phenotype association. The HPO Goodness of Fit (HGF) score is then calculated from each heatmap which allows for prioritisation of genes per phenotype. In addition, the heatmap is also used to derive a predicted mode of inheritance (MOI) of a gene-phenotype relation, which is a common use case when a novel gene is under consideration for a patient with unknown family history.

We applied Phenogenon to the Phenopolis exome dataset and were able to recapitulate known gene-phenotype relations, such as "*SRD5A3*—Abnormal full-field electroretinogram—recessive" and "*GRHL2* –Nail dystrophy—recessive". We also discovered potentially a novel relation, "*RRAGA*–Abnormality of the skin—dominant".

Scripts to perform Phenogenon analysis are available at https://github.com/phenopolis/phenogenon and an interactive visualisation tool is available at https://phenogenon.phenopolis.org.

## Materials and methods

### Patient phenotyping and selection

This study dataset includes 5122 exomes from the Phenopolis database comprising Mendelian and common disease patients. We used Human Phenotype Ontology [11] (HPO) as the standardised phenotype vocabulary for recording patient phenotypes, which were entered manually from patient notes by medical coders and extracted computationally from patient letters using cTAKES [12]. Patient relatedness was estimated using KING [13], and related individuals were excluded so as not to skew the genetic association tests. This resulted in a subset of 3290 exomes from unrelated individuals (Table 1).

**Table 1:** Total number of 3290 exomes by predominant phenotypes.

Predominant phenotype(s)	Number of samples
Dementia (with relation to prion disease)	1039
Inflammatory bowel disease	653
Retinal disorders	504
Healthy	272
Epilepsy	241
Bone Marrow Failure	190
primary immunodeficiency	109
Sudden Cardiac Death	92
Mitochondrial diseases	89
Dermotological disorders	47
Arrhythmogenic right ventricular cardiomyopathy	27
Nervous system disorders	14
Cataract	5
Mitochondrial diseases	4
Keratoconus	4

### Variant calling and filtering

The variant calling and annotation pipeline has been described previously [10]. In brief, exomes were aligned using Novoalign to build GRCh37 of the human genome and variants were called and filtered using the Genome Analysis Tool Kit (GATK) best practices. Variants that did not pass the GATK filters, were not covered in gnomAD or were non-coding, defined as more than 5 base pairs away from nearest coding region, were filtered out. Variants with a missing rate of more than or equal to 20% in our data were also discarded. This left a total of 973,426 variants which were annotated with gnomAD frequencies [14] and CADD Phred score [15]. GnomAD was used as it remains the largest resource for population level variant frequency annotation; and CADD due to its popularity, ability to predict indels and ease of local installation.

### Scoring “gene—Phenotype—Mode of inheritance” associations

We considered variant frequencies in gnomAD under both modes of inheritance (MOI), dominant or recessive. In the case of dominant inheritance, we defined the variant gnomAD frequency (GF) to be the gnomAD allele frequency, and in the case of recessive inheritance, the estimated homozygote frequency:

$$GF\;=\;\{\begin{array}{ll} \frac{AC}{AN}, & if\;recessive\\ \frac{2\times HOM}{AN}, & if\;dominant \end{array}$$

**Fig 1:**
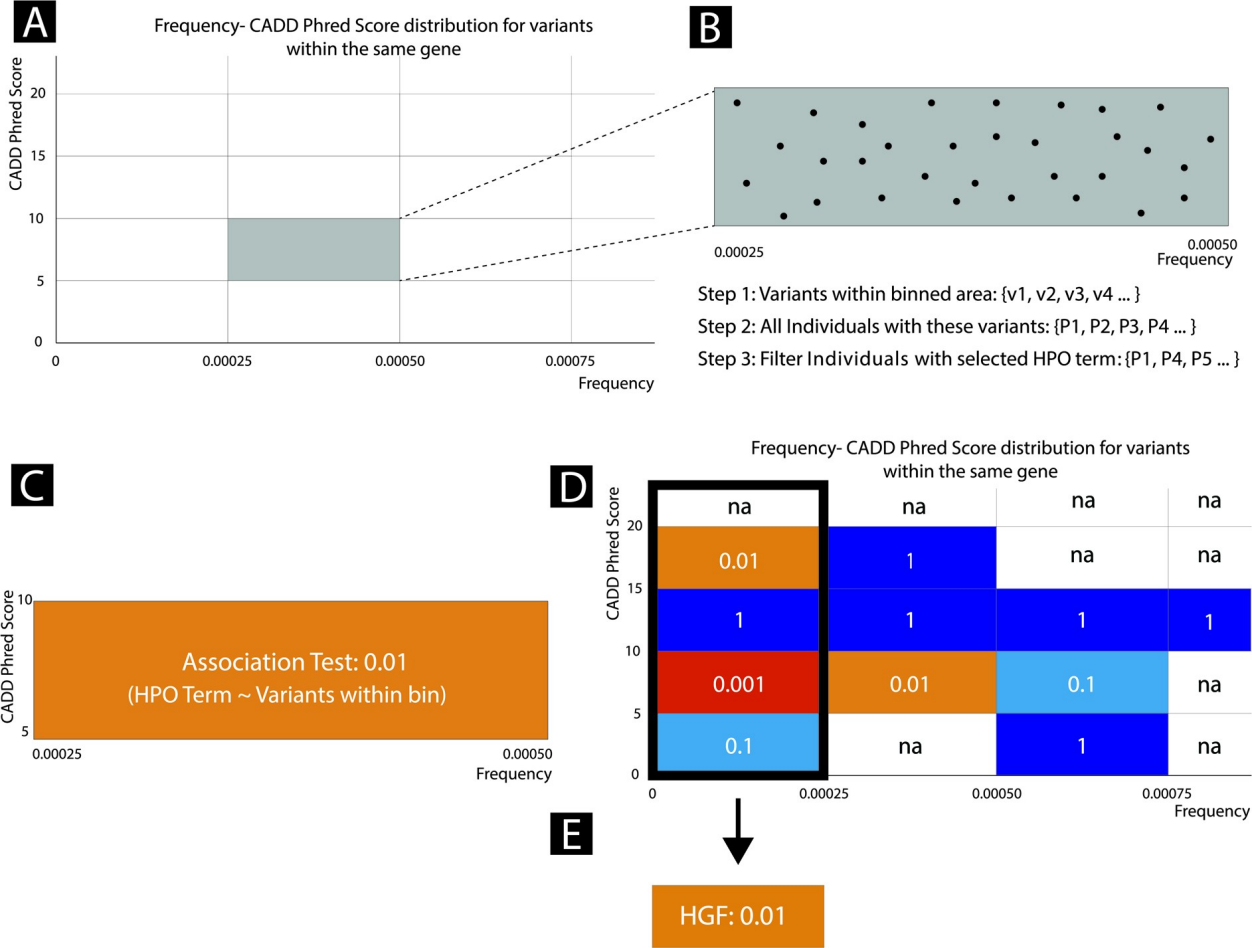
Phenogenon profiling workflow. A) The distribution of frequency vs CADD Phred score for variants of a single gene were binned according to empirically chosen cut-offs. B) Variants within each binned area are further analysed. Individuals carrying these variants are identified and then filtered on the basis of whether they have a selected HPO term. C) Fisher’s Exact test is then used to determine the significance of the gene-phenotype relationship. D) A Phenogenon heatmap is produced using the Fisher Exact P-Values for each binned area. E) Fisher Exact Scores for each of the binned area in the first column are collapsed into a single HPO goodness of fit score (HGF) using a Scaled Stouffer transformation.

Given a gene, HPO term and MOI, variants found on the gene are binned according to their GF and CADD score (Fig 1A and 1B). We selected a bin height of 5 for CADD and a bin width of 1/4000 = 0.00025 for GF. From here on, variants with a GF < 0.00025 are considered to be rare variants. Binned variants are then used to identify patient carriers who are considered to be either cases or controls based on whether they had the selected HPO term or any of its child terms (Fig 1B). A case/control Fisher’s exact test (Fig 1C) is applied to each bin according to the contingency table in S1 Table. The Fisher test is repeated for all bins and a heatmap is produced coloured by the negative logarithm of the p-values. This heatmap is referred to as the Phenogenon profile for a “gene—HPO—MOI” relationship (Fig 1D). The z scores of the bins are then weighted (*w*_*i*_), according to S2 Table, and summed using a variation of Stouffer’s Z-score method, in order to obtain an overall Z score for the “gene—HPO—MOI” relationship (Fig 1D and 1E):

$$Z∼\frac{\sum_{i\;=\;1}^{k}{w_{i}Z}_{i}}{\sqrt{k_{rare}}}$$ (1)

Where k_rare_is the number of non-empty rare bins (GF < 0.00025). The motivation for the scale factor k_rare_ is explained in the S1 File. Finally, the Z score is converted to a p-value assuming a standard normal distribution and the negative logarithm of the p-value is used to define the HPO Goodness of Fit (HGF) for that “gene—HPO—MOI” relationship:

HGF=-log(1-ϕ(z)) (2)

Where ϕ is the cumulative density function of the normal distribution.

For a given gene and HPO term, HGF scoring can be performed assuming either dominant or recessive mode of inheritance (MOI). When testing for recessive MOI, patients are assumed to be compound heterozygous if they carry a second variant, with a higher or equivalent CADD score and a lower or equivalent GF.

The signal ratio is calculated for each “HPO-gene-MOI” relationship, based on the observation that if a wrong MOI is assumed, the Phenogenon heatmap profile tends to produce more significant p-values bins for non-rare variants (GF > 0.00025) (S2 Fig).

The signal ratio (SR) is defined as:

$$SR\;=\;\frac{\sum_{i\;=\;1}^{k_{rare}}-ln(p_{i})}{\sum_{j\;=\;1}^{k_{all}}-ln(p_{j})}$$ (3)

Where k_all_ represents the total number of non-empty non-rare bins with GF > 0.00025.

The “gene-HPO-MOI” score is then defined as:

M={HGFrec×SR,ifrecessiveHGFdom×SR,ifdominant (4)

The larger M value is deemed to be the most likely MOI.

### Benchmarking Phenogenon

In order to benchmark our method and to choose an appropriate NP and HGF cut-off, we selected a list of 12 known gene-HPO-MOI relationships (Table 2). Our list included *SCN1A* (for dominant MOI) and *ABCA4* (for recessive MOI). *SCN1A* encodes Sodium Voltage-Gated Channel Alpha Subunit 1, mutations of which have been linked to epilepsy with divergent clinical severity [16]. The mutations are either dominantly inherited or arise *de novo* [17] with the majority of mutations found in the severe form of epilepsy (severe myoclonic epilepsy in infancy; MIM# 607208) being mostly *de novo* [16]. *ABCA4* encodes ATP Binding Cassette Subfamily A Member 4, and biallelic mutation of the *ABCA4* gene leads to a spectrum of retinal diseases including Stargardt macular dystrophy, and cone-rod dystrophy [18].

**Table 2:** Known HPO-gene-MOI relationships used to benchmark Phenogenon.

Rank	Gene	HPO	MOI	NP	M score	HGF score
1	*SCN1A*	Seizures	Dom	100	Dom	64.43
2	*USH2A*	Visual impairment	Rec	259	Rec	26.20
3	*ABCA4*	Macular dystrophy	Rec	76	Rec	16.78
4	*CNGB1*	Constriction of the peripheral visual field	Rec	41	Rec	9.43
5	*CERKL*	Nyctalopia	Rec	15	Rec	8.25
6	*PROM1*	Macular dystrophy	Dom	60	Dom	7.02
7	*GUCY2D*	Visual loss	Rec	8	Rec	6.82
8	*CRB1*	Retinal dystrophy	Rec	25	Rec	6.75
9	*TERT*	Bone marrow hypocellularity	Dom	48	Dom	6.28
10	*BBS1*	Constriction of the peripheral visual field	Rec	10	Rec	5.61
11	*RPGR*	Constriction of the peripheral visual field	X-linked	28	Rec	4.77
12	*IMPG2*	Visual loss	Rec	4	Rec	2.51

MOI = Mode of Inheritance; NP = the number of patients who carry rare variants for the corresponding MOI

We also compared the performance using our Phenogenon modified Stouffer’s Z-score method compared to Fisher’s method. Similar to Stouffer’s Z-score method, Fisher’s method also combines p-values to produce an overall p-value. However, it lacks the ability to assign weights, and therefore treats bins with different CADD phred scores equally. Specifically, Fisher’s method combines p-values using the following formula:

$$X_{2k}^{2}\sim -2\sum_{i\;=\;1}^{k}ln(p_{i})$$

Where *X*^*2*^ is a test statistic that follows a chi-squared distribution.

For each gene, we determined the MOI (using the M score) for each of the HPO terms with an affected sample size > = 60, unless stated otherwise; then according to the determined MOI, we calculated an HGF score for each of the HPO term. We calculated a mean and a standard deviation of the HPO HGF scores for the gene, and chose HPO terms with an HGF score at least one standard deviation higher than the mean as positive hits for the gene. We then compare the positive HPO terms with a set of hand-curated truth set to determine an error rate.

We benchmarked Phenogenon on predictions for the HPO terms and the MOI for each gene. A gene-HPO relation is deemed true if the relation is supported by the Human Phenotype Ontology.

We surmised that Phenogenon would not perform well for HPO terms that are too specific or too general. Specific HPO terms have small number of affected patients (NP), which limit the power of any measures of association analysis. On the other hand, general HPO terms, such as ‘Phenotypic abnormality’ (HP:0000118) and ‘All’ (HP:0000001), include almost all the samples for test, and will limit the analysis power in a similar way. To find out the optimal sample sizes for predictions, we chose a number of NP cut-offs to choose to only predict HPO terms with a NP equal or higher than the cut-offs.

We surmised that MOI prediction works best for gene-HPO relations supported with a high HGF score. To assess MOI predictions, we first chose an HGF cut-off, and benchmark MOI prediction on gene-HPO relations with a HGF score higher than the HGF cut-off. For comparison, we chose to use HGF score only for MOI prediction, so that:

MOI={dominantifHGFd>HGFrrecessiveifHGFd<HGFrundefifHGFd=HGFr

Where HGF_d_ and HGF_r_ are HGF scores assuming dominant and recessive MOI, respectively.

To demonstrate the benefit of using estimated homozygote frequency over allele frequency for association analyses when assuming recessive MOI, we also included predictions for comparison to use allele frequency (instead of estimated homozygote frequency) to produce M and HGF scores for recessive relations.

### Phenogenon on a large patient cohort

Following the benchmarking, we applied Phenogenon to all protein coding genes in the Phenopolis dataset (number of unrelated patients: 3290, number of protein coding genes with variants: 21321), under both dominant and recessive inheritance modes. A breakdown of patient phenotypes is shown in Table 1.

## Results

### Phenogenon made correct predictions on both HPO and MOI in a controlled environment

To benchmark Phenogenon, we selected 12 genes for which mutations have been reported to be causal in the cohort. The HPO term with highest HGF score for each tested gene can be found in Table 2.

**Fig 2:**
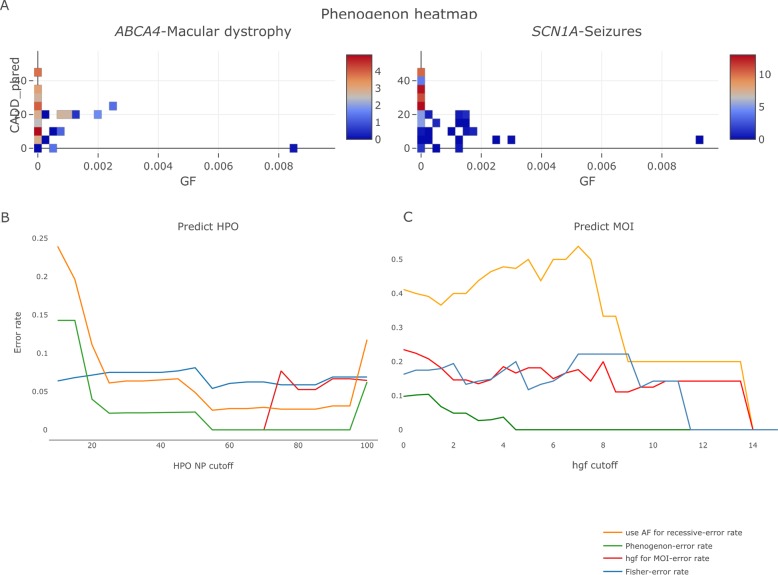
Using phenogenon to predict gene-HPO-mode of inheritance (MOI) relationships for the 12 known genes. A. Examples of using Phenogenon to profile known relationships: *ABCA4*—Macular dystrophy (HP:0007754) -recessive, and *SCN1A*—Seizures (HP:0001250)—dominant. The color scales represent the HGF score. The majority of high-scoring bins are for rare variants (HGF < 0.00025). B. Error rate in predicting HPO when number of patients selected per gene is higher than ‘HPO NP cut-off’. The lines give the trend of error rates for each prediction model. C. Error rate for MOI when HPO selected per gene is higher than HGF cut-off. The lines give the trend of error rates for each prediction model. Orange line: model using gnomAD allele frequency instead of estimated homozygote frequency for recessive MOI; Red line: model using HGF for both HPO association and MOI prediction; Blue line: model using Fisher method to combine p values; Green line: our current model for Phenogenon.

As shown in Fig 2A, for both “*ABCA4 –*Macular dystrophy—recessive” and “*SCN1A –*Seizures—dominant”, bins showing strong association correctly clustered with rare variants (GF < 0.00025).

Phenogenon (green line, Fig 2B) outperformed Fisher (blue line, Fig 2B), demonstrating the benefit of assigning higher weights to bins with higher CADD score.

Phenogenon correctly predicted HPO terms for which there are at least 55 patients affected (NP > 55) (green line, Fig 2B), although as expected, the error rate increased when including HPO terms see in fewer patients (NP < 20). Interestingly, the error rate increased when HPO NP > 100 (Fig 2B), suggesting that there are divergent genetic causes for less specific HPO terms. In addition, it also made wrong HPO prediction when assuming wrong MOI.

The M score (green line, Fig 2C) was more accurate in predicting the MOI than using HGF alone (red line, Fig 2C). Furthermore, as shown in Fig 2B and 2C, using GF defined as the gnomAD allele frequency when assuming recessive MOI (orange line) had a poorer performance than using estimated homozygote frequency (green line) in predicting HPO and MOI.

### Phenogenon found known gene-HPO-MOI relationships in a large patient cohort

We performed Phenogenon on the 3290 unrelated samples of the Phenopolis cohort. As shown in Table 3, from the top 10 relations discovered using Phenogenon, six were known (*SCN1A* and *USH2A* are shown in Table 2 instead); the MOI of all were predicted correctly. We were also able to uncover other strong gene-phenotype relationships when including HPO terms with at least 60 individuals affected (Table 3). For instance, *GRHL2* (OMIM: 608576) known to cause recessive ectodermal dysplasia/short stature syndrome, which involves nail dystrophy [19], was correctly linked to Nail dystrophy with recessive MOI by Phenogenon (HGF score: 10.54). *STAT1* (OMIM: 600555) known to cause dominant or recessive immunodeficiency, was also correctly linked to Severe combined immunodeficiency, with dominant MOI (HGF score: 10.38). Other examples include *SRD5A3 –*Abnormal full-field electroretinogram (HGF: 11.13) with recessive MOI (known to cause recessive congenital disorders of glycosylation, which may involve retinal disorders [20].) and *PDE6A –*Retinal dystrophy (HGF: 9.40) with recessive MOI (known to cause recessive retinitis pigmentosa [21]). Among the top 10 findings, there are four relations that were previously unreported. Whilst three of them were likely false positives, we think that the association of “*RRAGA—*abnormality of the skin—dominant” may reflect a novel disease mechanism. *RRAGA* encodes Ras-related GTP-binding protein A that activates mTORC [22], which was found to regulate skin morphogenesis and epidermal barrier formation [23], therefore its mutations are the possible pathogenic cause of the skin disorders observed on the patients in the Phenopolis dataset. *AIP* encodes a receptor for aryl hydrocarbons and a ligand-activated transcription factor, and was associated with Dementia by Phenogenon. This is likely a false positive since all the variants contributing to the HPO’s high HGF score had low sequencing depths (2 to 7) and were all called as homozygote by GATK. Given that the gnomAD allele frequencies of the variants are zero, the likelihood of observing multiple homozygous carriers of the variants in our unrelated samples is low. Considering their low alignment depths, they are likely genotyping errors. *NUP205* encodes a nucleoporin, and was associated with Abnormal electroretinogram by Phenogenon. On the other hand, majority of the variants in the low p value bins in “*NUP205* –Abnormal electroretinogram—dominant” have a GF higher than 0. This contradicts the presumption that most retinal disorders in the Phenopolis dataset are rare Mendelian disorders, therefore we believe “*NUP205* –Abnormal electroretinogram—dominant” is a false positive. Interestingly, despite that experts in the consortium have ruled out *TTN* as a causative gene for retinal disorders, the reason why Phenogenon associated *TTN* with Abnormality of the anterior segment of the globe remains unclear.

**Table 3:** Top-ranked gene-phenotype-MOI relations reported by phenogenon.

Gene	Gene Description	Predicted HPO	Predicted MOI	Known MOI	HGF score	Known
*RRAGA*	Encodes Ras-related GTP-binding protein A that activates Mtorc [22], which regulates skin morphogenesis and epidermal barrier formation [23].	Abnormality of the skin	dominant	/	11.43	No
*SRD5A3*	Steroid 5α-reductase type 3 is known to cause congenital disorders of glycosylation, which may involve retinal disorders [20].	Abnormal full-field electroretinogram	recessive	recessive	11.13	Yes
*AIP*	Known to cause pituitary adenoma [24]	Dementia	recessive	/	11.03	No
*NUP205*	*NUP205* encodes a nucleoporin, known to cause steroid-resistant nephrotic syndrome [25].	Abnormal electroretinogram	recessive	/	10.98	No
*GRHL2*	Transcription factor involved in multiple cancers and keratin development [19,26],	Nail dystrophy	recessive	recessive	10.54	Yes
*STAT1*	Gain of function variants in this transcription factor exhibit diverse immune dysfunction [27,28]	Severe combined immunodeficiency	dominant	dominant/recessive	10.38	Yes
*TTN*	Involved in cardiomyopathy [29]. Very large gene prone to artefacts [30].	Abnormality of the anterior segment of the globe	dominant	/	9.74	No
*PDE6A*	*PDE6A* expresses in cells of the retinal rod outer segment, and is known to cause retinitis pigmentosa [21].	Retinal dystrophy	recessive	recessive	9.40	Yes

## Discussion

Aggregated databases of high throughput sequencing data of large numbers of HPO-annotated patients are indispensable for the genetic diagnosis of rare disease patients.

However, phenotypic and genetic biases are often inherent to these datasets. Phenotypic bias may be caused by certain patients such as in our dataset, eye patient, having more HPO terms than other types of patients, such as neurological patients, that typically only have one HPO term. Genetic bias may be caused by exome capture biases in coverage which we attempted to control for by imposing strict thresholds on the missingness. Despite these phenotypic and genetic biases, using our new tool, Phenogenon, we were able to recapitulate several known gene-phenotype-MOI relationships (Table 3).

Phenogenon can also be applied to a combination of phenotype terms. For example, considering patients affected by both ‘Rod-cone dystrophy’ (HP:0000510) and ‘Hearing impairment’ (HP:0000365), the top two genes predicted are *USH2A* (HGF: 9.53) and *ADGRV1* (HGF: 8.31), both known to cause Usher syndrome that affects both visual and hearing systems. However, a caveat of such an approach is a reduced sample size hence decreased predictive power.

We recognise our reported novel relations require further scrutiny, in particular in the case of dominant MOI associations, as the results of these are sensitive to various parameters such as the version of CADD used. In particular, we witnessed CADD score increases for a number of synonymous variants between version 1.3 and 1.4 of CADD. Furthermore, the association signal can also be driven by uncharacteristic variants with a higher GF and CADD than expected. For instance, in the predicted relation “*NUP205*—Abnormal electroretinogram—dominant”, around 70% of the enriched rare variants have GF > 0 while having a CADD > 15 (S3 Fig). The “*TTN*–Abnormality of the anterior segment of the globe—dominant” also warrants further investigation as this is a large gene prone to artefact (S4 Fig). We therefore recommend that these relationships are examined more closely using our interactive webtool https://phenogenon.phenopolis.org.

Until the release of the gnomAD database, there was no reliable source to estimate variant homozygote frequency, and therefore to date, all gene-phenotype association tools have used allele frequency, regardless of the MOI. We argue that using homozygote frequency when assuming recessive MOI improves the model performance.

In conclusion, we have developed a statistical tool, Phenogenon, to detect and visualise “gene—HPO—MOI” relationships. Our approach has suggested some strong candidate relationships and correctly recapitulated existing relationships. The adoption of the HPO nomenclature by large rare disease sequencing projects leads us to believe Phenogenon will be of increasing utility in understanding gene-phenotype-MOI relationships as genetics is phased into routine NHS practice.

## Supporting information

S1 File (DOCX)

S1 Fig (TIFF)

S2 Fig (TIFF)

S3 Fig (TIF)

S4 Fig (TIF)

S1 Table (DOCX)

S2 Table (DOCX)

## Abbreviations

CADD: Combined Annotation Dependent Depletion
GF: Gnomad Frequency
HGF: HPO Goodness of Fit
MOI: Mode of Inheritance
NP: Number of Patients
SR: Signal Ratio
