# Supplementary material for: Phenogenon: Gene to phenotype associations for rare genetic diseases
Source: PLoS One. 2020 Apr 9;15(4):e0230587. doi: 10.1371/journal.pone.0230587 (PMC7144978; doi:10.1371/journal.pone.0230587)
Supplement: S1 File — (DOCX) [file pone.0230587.s001.docx]

Stouffer's method to combine p-values from multiple tests is calculated as follows:

$Z\sim\frac{\sum_{i=1}^{k} {w_{i}Z}_{i}}{\sqrt{\sum_{i=1}^{k} w_{i}^{2}}}$

However, Z scores are difficult to compare across gene-phenotype associations if few bins contribute to the score, which is more likely for smaller genes with fewer variants. For example, consider two HPO-gene relationships *l* and *h* where only one bin contributes to the Z score in each case, then these will yield the same overall Z score as the weights cancel out:

$\frac{w_{l}Z}{\sqrt{w_{l}^{2}}}=\frac{w_{h}Z}{\sqrt{w_{h}^{2}}}$

In order to account for this and make Z scores comparable across genes, we redefine the Z score as follows:

$Z\sim\frac{\sum_{i=1}^{k} {w_{i}Z}_{i}}{\sqrt{k_{rare}}}$

Where k_rare_ is the number of non-empty rare bins (GF < 0.00025).
