# Supplementary figures and images for: Phenogenon: Gene to phenotype associations for rare genetic diseases
Source: PLoS One. 2020 Apr 9;15(4):e0230587. doi: 10.1371/journal.pone.0230587 (PMC7144978; doi:10.1371/journal.pone.0230587)

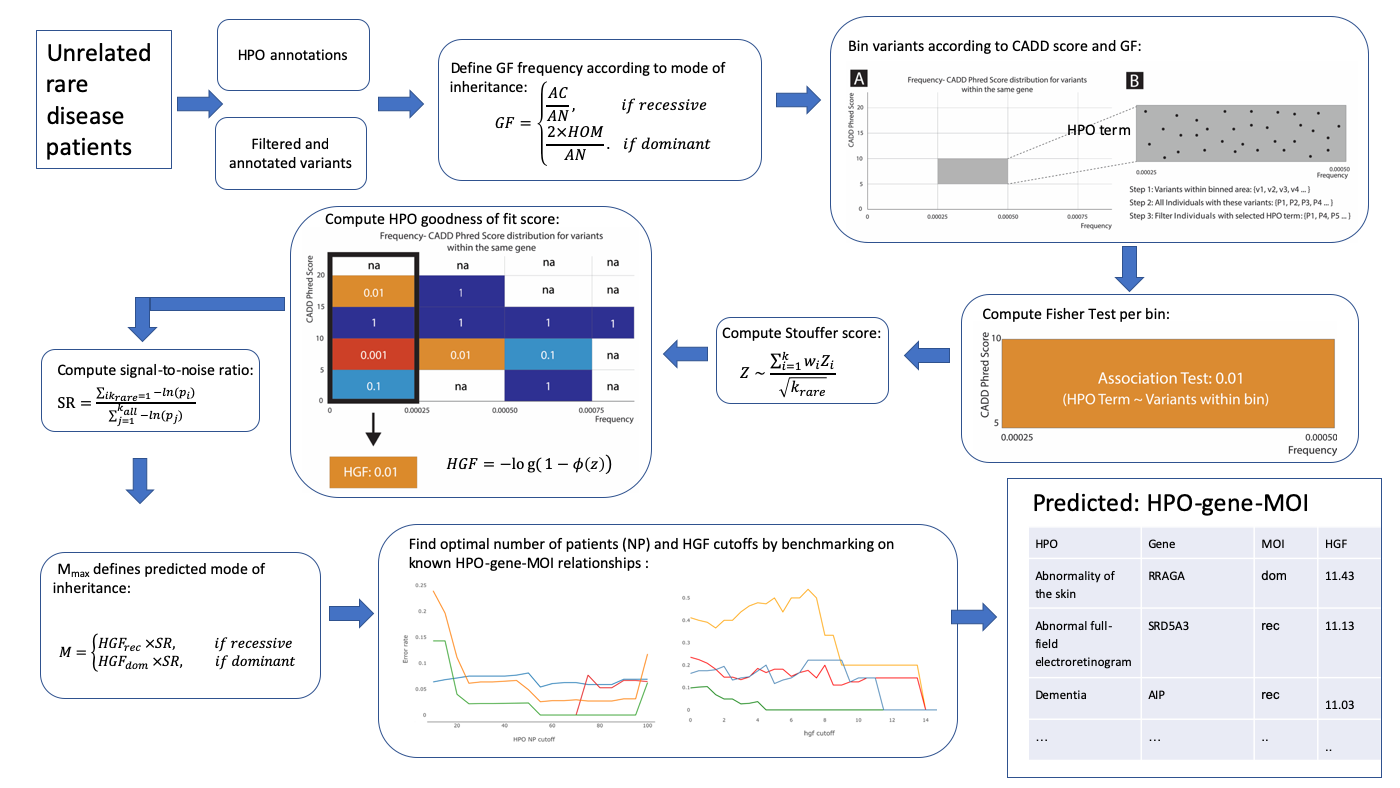

Supplement: S1 Fig — (TIFF) [file pone.0230587.s002.tiff]

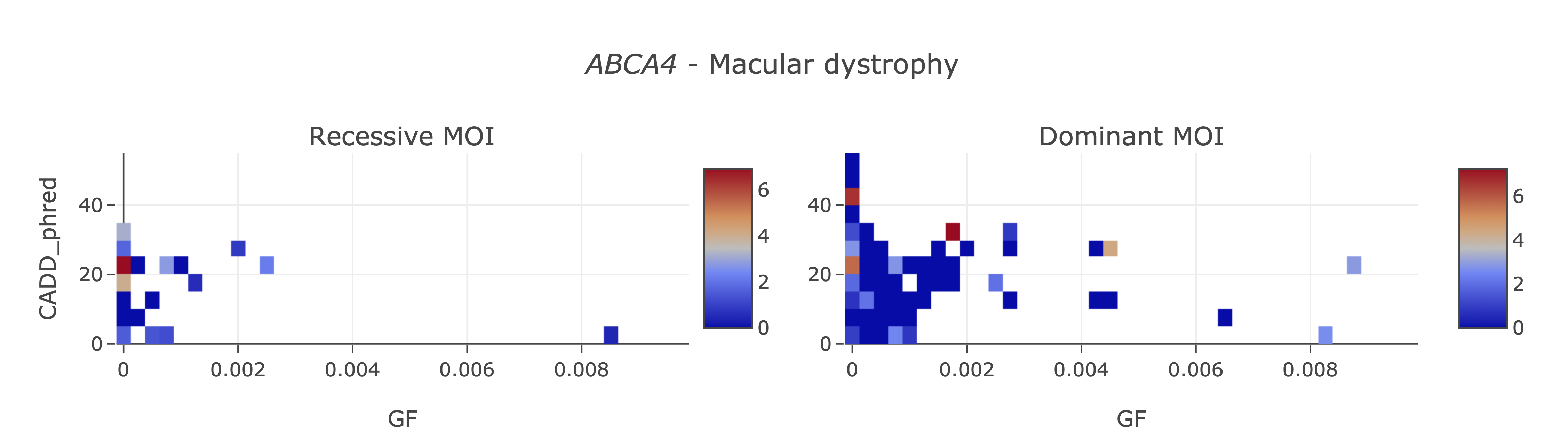

Supplement: S2 Fig — (TIFF) [file pone.0230587.s003.tiff]

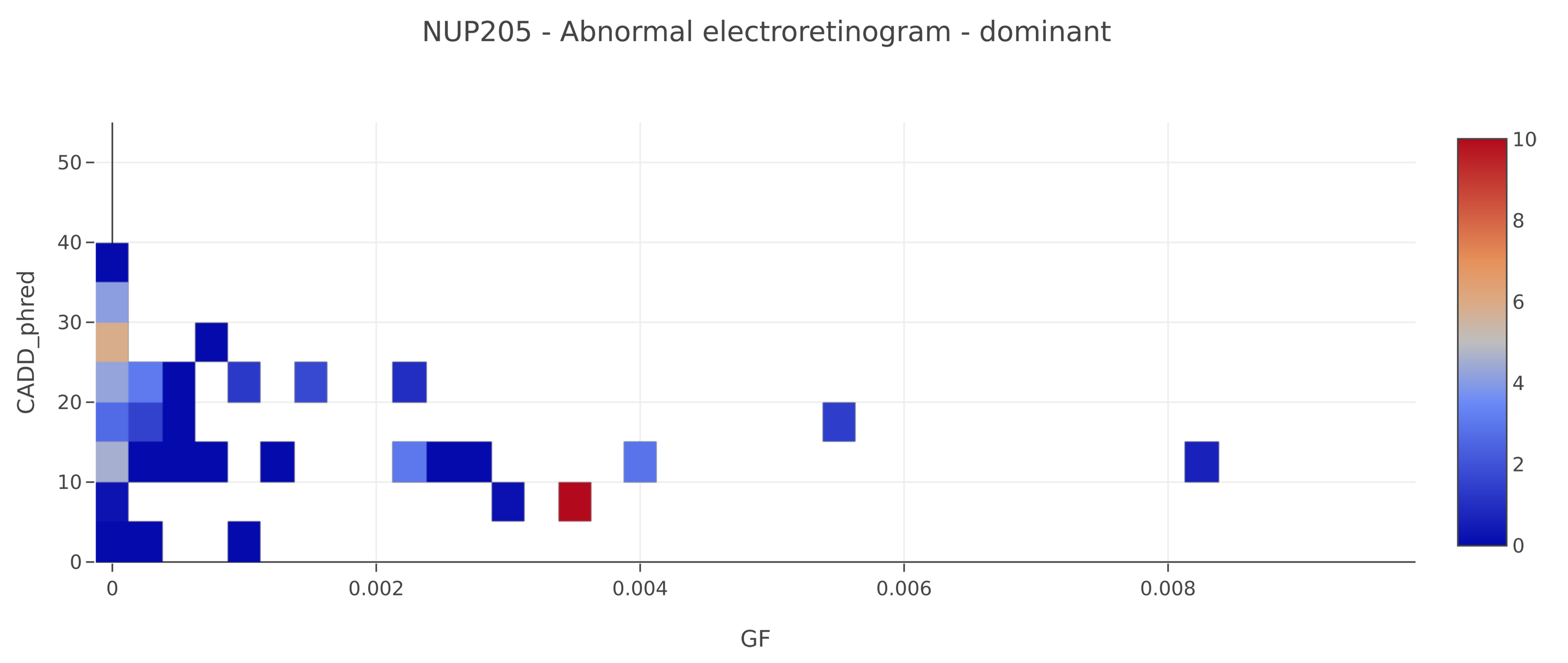

Supplement: S3 Fig — (TIF) [file pone.0230587.s004.tif]

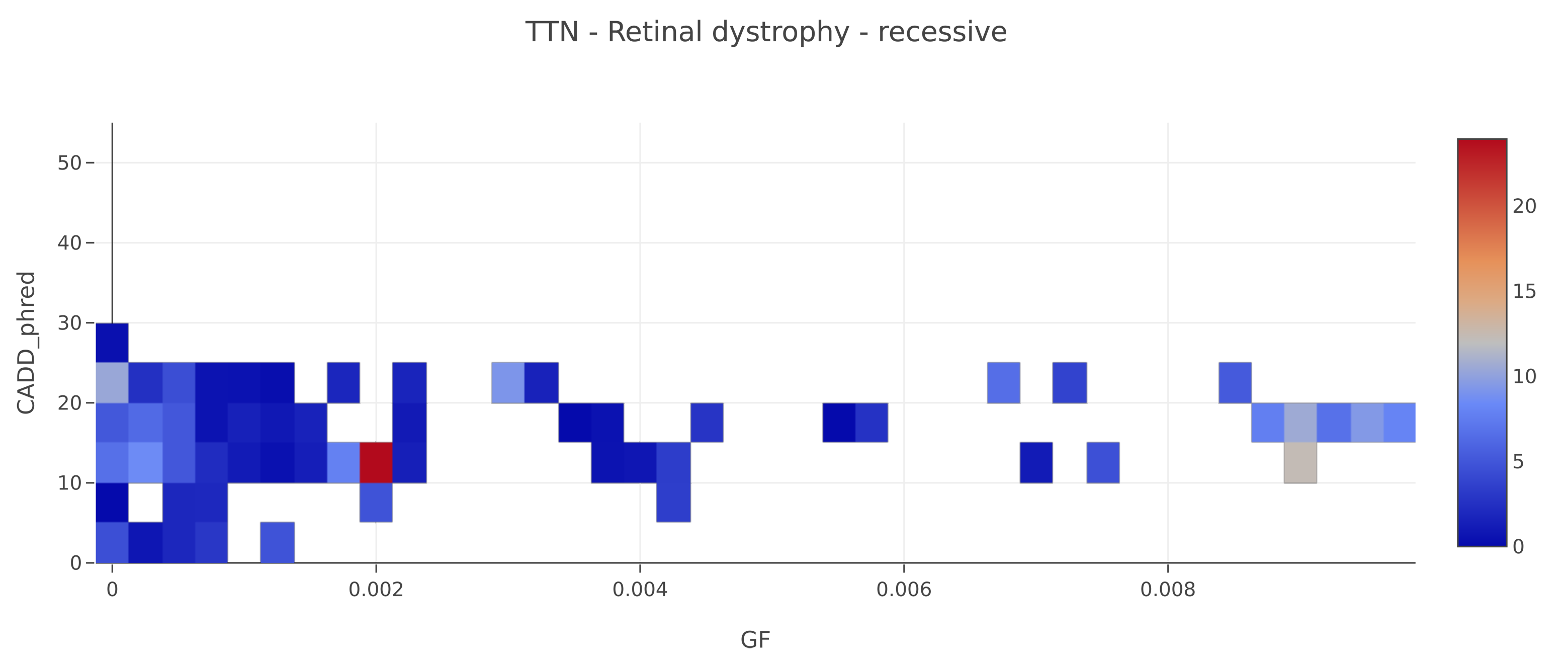

Supplement: S4 Fig — (TIF) [file pone.0230587.s005.tif]
