# Supplementary material for: Phenogenon: Gene to phenotype associations for rare genetic diseases
Source: PLoS One. 2020 Apr 9;15(4):e0230587. doi: 10.1371/journal.pone.0230587 (PMC7144978; doi:10.1371/journal.pone.0230587)
Supplement: S1 Table — (DOCX) [file pone.0230587.s006.docx]

|  | Patients without the HPO term | Patients with the HPO term |
| --- | --- | --- |
| Patients not carrying a variant in bin k |  |  |
| Patients carrying a variant in bin k |  |  |
