# Supplementary material for: Phenogenon: Gene to phenotype associations for rare genetic diseases
Source: PLoS One. 2020 Apr 9;15(4):e0230587. doi: 10.1371/journal.pone.0230587 (PMC7144978; doi:10.1371/journal.pone.0230587)
Supplement: S2 Table — (DOCX) [file pone.0230587.s007.docx]

| **CADD** |  | | | |  |
| --- | --- | --- | --- | --- | --- |
| [55,60) | 1.8 | 0 | 0 | 0 |  |
| [50,55) | 1.7 | 0 | 0 | 0 |  |
| [45,50) | 1.6 | 0 | 0 | 0 |  |
| [40,45) | 1.5 | 0 | 0 | 0 |  |
| [35,40) | 1.4 | 0 | 0 | 0 |  |
| [30,35) | 1.3 | 0 | 0 | 0 |  |
| [25,30) | 1.2 | 0 | 0 | 0 |  |
| [20,25) | 1.1 | 0 | 0 | 0 |  |
| [15,20) | 1.0 | 0 | 0 | 0 |  |
| [10,15) | 0.75 | 0 | 0 | 0 |  |
| [5,10) | 0.5 | 0 | 0 | 0 |  |
| [0,5) | 0.1 | 0 | 0 | 0 |  |
|  | [0,0.00025) | [0.00025,0.0005) | [0.0005, 0.00075) | [0.00075,1.0) | **GF** |
